# Isolation and Characterization of New Microsatellite Markers for the Invasive Softshell Clam, *Mya arenaria* (L.) (Bivalvia: Myidae)

**DOI:** 10.3390/ijms13022515

**Published:** 2012-02-22

**Authors:** Ana-Maria Krapal, Oana Paula Popa, Elena Iulia Iorgu, Marieta Costache, Luis Ovidiu Popa

**Affiliations:** 1 Molecular Biology Department, “Grigore Antipa” National Museum of Natural History, Kiseleff Street, No 1, Bucharest 011341, Romania; E-Mails: ana.krapal@antipa.ro (A.-M.K.); oppopa@antipa.ro (O.P.P.); elenap@antipa.ro (E.I.I.); 2 Departments of Biochemistry and Molecular Biology, University of Bucharest, 91-95 Spl. Independentei, Bucharest 050095, Romania; E-Mail: marietacostache@yahoo.com; 3 Faculty of Biology, Alexandru Ioan Cuza University of Iasi, 22, Carol I Boulevard, Iaşi RO 700505, Romania

**Keywords:** genetic diversity, invasive, population genetics, Black Sea

## Abstract

The invasive softshell clam (*Mya arenaria* Linnaeus, 1758) is native to the northwestern region of the Atlantic Ocean. This species has been introduced in the northeast Pacific and along the European coasts, due to intense naval transports and aquaculture, and it is now present in all the European seas. In this paper we describe seven new microsatellite loci for *Mya arenaria*. The isolated loci are polymorphic with a number of alleles per locus between 6 and 14. The observed and expected heterozygosities ranged from 0.417 to 0.951, and from 0.643 to 0.895, with an average of 0.716 and 0.775, respectively. These microsatellite markers should be useful in analyzing this species’ genetic diversity, which could explain various processes of its invasion history.

## 1. Introduction

*Mya arenaria* is one of the most important commercial clams in the USA, being used as a food source [1]. Its current distribution includes the northwest Atlantic (from Nova Scotia to Virginia), the northeast Pacific (from San Francisco to Alaska) and the European seas (North, Black, Baltic, Mediterranean, Waden and White Seas) and is similar to the distribution of the species before the Last Glacial Maximum. During the Pleistocen glaciation, it is thought that all the softshell clam populations died out, except the one from the north-west of the Atlantic [2]. After this event, *M. arenaria* has reinvaded the eastern regions of the Atlantic, most likely as a result of human activity, and it is thought that the re-invasion of the European coasts has begun somewhere in the thirteenth century as a result of Viking expeditions [3]. The softshell clam was recorded for the first time in the Black Sea in 1966 in Odessa Bay and, since then, it has spread rapidly on Romanian and Ukrainian coasts [2].

Genetic population studies on invasive species are useful in obtaining information about the geographic origins of invasive populations, the route(s) of invasion, number of introductions, and levels of genetic diversity and gene flow. Lately, DNA microsatellite markers have become the most utilized tool in studying such processes. However, the microsatellite loci available for molluscs species are still limited [4] and until now, studies of genetic variation on *M. arenaria* were conducted using only allozymes [5] and COI sequences [6]. Recently, 8 microsatellite loci for this species have been described [7]. Computer simulations showed that over 30 microsatellite loci should be used when inferring aspects of the evolutionary history of populations, [8] therefore, additional microsatellite loci are needed for population genetic studies. In this paper we describe another seven new highly polymorphic microsatellite loci which can also be used in analyzing the genetic variability of *Mya arenaria*.

## 2. Results and Discussion

All of the sequenced clones contained repetitive motifs, but only 25 of them were suitable for primer design. Out of the 25 primer pairs, only 7 gave consistent amplification and were subsequently used for polymorphism screening (Table 1).

The number of alleles per locus ranged from 6 to 14, with an average of 8.9. The observed and expected heterozygosities ranged from 0.417 to 0.951, and from 0.643 to 0.895, with an average of 0.716 and 0.775, respectively (Table 1). Significant deviation from Hardy-Weinberg equilibrium was observed after the Bonferroni correction, in 5 out of 14 (36%) possible single exact locus tests (*p* < 0.05). The results of the Micro-Checker testing showed that three loci (Ma02, Ma06, Ma12) exhibited an excess of homozygotes, most likely due to the presence of null alleles. While some authors reported these phenomena (HW deviation, null alleles) as common for bivalve species [9,10], we also notice that some loci may exhibit HW deviation in some population, but not in others. In our case, Ma02 exhibited HW deviation only in the Constanta (CT) population, while the Ma11 locus exhibited HW deviation only in the Mangalia (MG) population. No significant linkage disequilibrium was found between all pairs of these 7 loci after Bonferroni’s correction. While some authors reported a positive correlation between the length of the microsatellite repeat motif and the number of alleles for the respective loci [11], we did not find such a correlation in our data.

## 3. Experimental Section

Genomic DNA was extracted from the adductor muscle of a single *Mya arenaria* specimen using a phenol-chloroform protocol optimized for molluscs [12]. We isolated the microsatellite loci using a modified enrichment protocol [13,14]. The microsatellite enriched genomic library was screened for inserts and we selected a number of 55 positive clones (the clones that generated two bands on agarose gel) which were further sequenced using the LICOR 4300L Genetic Analyzer.

A total of 76 individuals of *M. arenaria* were genotyped from two populations sampled in 2011: 35 individuals from Modern Beach, Constanţa, Romania (44°10'43.1"N, 28°39'35"E) and 41 individuals from Marină Beach, Mangalia, Romania (43°48'33.5"N, 28°35'13.8"E). All individuals were preserved in 96% ethanol. Total genomic DNA was extracted from muscle tissue using a Macherey-Nagel NucleoSpin^®^ Tissue kit, according to the producer’s specifications.

The PCR primers for each microsatellite locus were designed with the Primer3 software [15]. The PCR genotyping reaction was performed in a 10 μL total volume containing about 10 ng of DNA template, 10 mM Tris-HCl (pH 8.8 at 25 °C), 50 mM KCl, 0.08% (v/v) Nonidet P40, 2 mM MgCl_2_, each dNTP at 0.1 mM, each primer at 0.05 μM, 0.02 μM of IRD700 or IRD800 labeled M13 primer and 0.5 units of Taq DNA polymerase (Fermentas UAB, Vilnius, Lithuania). The temperature profile of the PCR reaction consisted of an initial denaturation step at 95 °C for 2 min followed by 30 cycles of denaturation at 95 °C for 30 s, annealing at a specific temperature for each locus (see Table 1 for details on each locus) for 30 s and extension at 72 °C for 30 s, followed by a final extension step at 72 °C for 5 min. The genotyping process was performed using SagaGT v. 3.1 software package.

GenAlEx 6.4 [16] was used to estimate the number of alleles per locus (Na), observed heterozygosity (Hobs) and expected heterozygosity (Hexp). Deviation from the Hardy-Weinberg equilibrium (HWE) was tested using the same software package. The presence of null alleles was tested using Micro-Checker (v. 2.2.3) [17] while linkage disequilibrium test was carried out using GenePop [18,19].

## 4. Conclusions

In addition to the previously described markers, these new seven highly polymorphic microsatellite markers are a useful resource for further studying the genetic diversity of native and invasive populations of *Mya arenaria*. Further studies could reveal the route of invasion(s) of this species in the Black Sea, explaining if the invasion was a step-by-step process (from previously established European populations), or the Black Sea populations of *Mya arenaria* were established directly from a North American source.

## Figures and Tables

**Table 1:** Primer sequences and characteristics of the nine microsatellite loci successfully amplified in *Mya arenaria*.

Marker	GeneBank accession no.	Repeat motif	Primer sequence	Size range	*T*_a_	*N*_A_		*H*_O_		*H*_E_		*P*_HW_	

						CT	MG	CT	MG	CT	MG	CT	MG
Ma02	JN850609.1	(CT)_14_(CT)_2_(CT)_2_	F: ggccctatatacccagcac	204–246	52								
			R: tgctgtctgagaagcctgtg			9	9	0,552	0,683	0,801	0,720	0,000 *	0,092
Ma06	JN850610.1	(TC)_25_	F: cctgacgggaataaaaacca	148–212	50								
			R: gactgacactggtaaacatttcg			6	7	0,655	0,417	0,721	0,644	0,383	0,918
Ma11	JN850612.1	(GA)_6_(GA)_25_	F: tttggctcagaccatgtcaa	158–198	52								
			R: cggcgagcacactgtactat			7	8	0,813	0,951	0,773	0,775	0,165	0,000 *
Ma12	JN850613.1	(CT)_25_	F: gacatggctgtagaagaaattagaa	198–376	50								
			R: ttgcaacctctttggaaaatg			14	13	0,765	0,711	0,895	0,838	0,900	0,258
Ma14	JN850614.1	(GA)_17_(GA)_6_	F: agcgtgctaagaatggccta	184–254	53								
			R: gcaatttttgaaagtccagagc			6	7	0,567	0,725	0,643	0,674	0,212	0,149
Ma15	JN850615.1	(GA)_26_	F: aagggagggagtgacatgaa	241–321	52								
			R: taccaaatcccacggtcatt			13	7	0,818	0,902	0,880	0,733	0,000 *	0,058
Ma26	JN850617.1	(GA)_6_(GA)_3_(GA)_16_(GA)_3_	F: gtgcttggttatggcgagtt R: gcacacattttattacgagtgtatga	224–306	53								
		(GA)_5_(GA)_3_(GA)_4_(GA)_11_											
		(GA)_3_(GA)_7_				9	9	0,697	0,771	0,787	0,804	0,007 *	0,001 *

*T*_a_, annealing temperature (°C); *N*_A_, number of alleles; *H*_O_, observed heterozygosity; *H*_E_, expected heterozygosity; *P*_HW_, Hardy-Weinberg probability test; CT, Constanta population; MG, Mangalia population;

* indicated deviation from Hardy-Weinberg equilibrium (*P* < 0.05) after Bonferroni’s correction.

## References

[1] Abraham B.J., Dillon P.L. (1986). Species Profiles: Life Histories and Environmental Requirements of Coastal Fishes and Invertebrates (Mid-Atlantic). Softshell clam. Biol. Rep.

[2] Strasser M. (1999). *Mya arenaria*—an ancient invader of the North Sea coast. Helgol. Meer.

[3] Petersen K.S., Rasmussen K.L., Heinemeier J., Rud N. (1992). Clams before Columbus?. Nature.

[4] Geist J., Rottmann O., Schröder W., Kühn R. (2003). Development of microsatellite markers for the endangered freshwater pearl mussel *Margaritifera margaritifera* L. (Bivalvia: Unionoidea). Mol. Ecol. Notes.

[5] Lasota R., Hummel H., Wolowicz M. (2004). Genetic diversity of European populations of the invasive soft-shell clam *Mya arenaria* (Bivalvia). J. Mar. Biol. Assoc. UK.

[6] Strasser C.A., Barber P.H. (2009). Limited genetic variation and structure in softshell clams (*Mya arenaria*) across their native and introduced range. Conserv. Genet.

[7] Barker F.K., Bell J.J., Bogdanowicz S.M., Bonatto S.L., Cezilly F., Collins S.M., Dubreuil C., Dufort M.J., Eraud C., Fuseya R. (2011). Permanent genetic resources added to molecular ecology resources database 1 June 2011–31 July 2011. Mol. Ecol. Resour.

[8] Koskinen M.T., Hirvonen H., Landry P.-A., Primmer C.R. (2004). The benefits of increasing the number of microsatellites utilized in genetic population studies: An empirical perspective. Hereditas.

[9] Hedgecock D., Li G., Hubert S., Bucklin K., Ribes V. (2004). Widespread null alleles and poor cross-species amplification of microsatellite DNA loci cloned from the Pacific oyster *Crassostrea gigas*. J. Shellfish Res.

[10] Imo M., Seitz A., Johannesen J. (2010). Distribution and invasion genetics of the quagga mussel (*Dreissena rostriformis bugensis*) in German rivers. Aquat. Ecol.

[11] Geist J., Geismar J., Kuehn R. (2010). Isolation and characterization of the first microsatellite markers for the endangered swan mussel *Anodonta cygnea* L. (Bivalvia: Unionoidea). Conserv. Genet.

[12] Sokolov E.P. (2000). An improved method for DNA isolation from mucopolysaccharide-rich molluscan tissue. J. Moll. Stud.

[13] Popa O.P., Iorgu E.I., Krapal A.M., Kelemen S.B., Murariu D., Popa L.O. (2011). Isolation and characterization of the first microsatellite markers for the endangered relict mussel *Hypanis colorata* (Mollusca: Bivalvia: Cardiidae). Int. J. Mol. Sci.

[14] Popa O.P., Popa L.O., Krapal A.M., Murariu D., Costache M., Iorgu E.I. (2011). *Sinanodonta woodiana* (Mollusca: Bivalvia: Unionidae): Isolation and characterization of the first microsatellite markers. Int. J. Mol. Sci.

[15] Rozen S., Skaletsky H, Krawetz S., Misener S. (2000). Primer3 on the WWW for General Users and for Biologist Programmers. Bioinformatics Methods and Protocols.

[16] Peakall R., Smouse P.E. (2006). GENALEX 6: Genetic analysis in Excel. Population genetic software for teaching and research. Mol. Ecol. Notes.

[17] Van Oosterhout C., Hutchinson W.F., Wills D.P.M., Shipley P. (2004). Micro-checker: Software for identifying and correcting genotyping errors in microsatellite data. Mol. Ecol. Notes.

[18] Rousset F. (2008). Genepop'007: A complete reimplementation of the Genepop software for Windows and Linux. Mol. Ecol. Resour.

[19] Raymond M., Rousset F. (1995). GENEPOP (version 1.2): Population genetics software for exact tests and ecumenicism. J. Heredity.

